# Supplementary material for: Certified nursing assistants’ perspectives on their role in advance care planning for older persons: A qualitative study
Source: Palliat Med. 2026 Jul 31;40(8):1158–67. doi: 10.1177/02692163261455222 (PMC13478406; doi:10.1177/02692163261455222)
Supplement: Supplementary file 1 [file sj-docx-1-pmj-10.1177_02692163261455222.docx]

**Supplementary file 1.** **Details about the participating organisations**

| **Participant** | **Organisation** | **Area** | **Setting** | **Number of community care teams**  **and residential care locations** |
| --- | --- | --- | --- | --- |
| 1 | 1 | Suburban | Community care | 45 community care teams |
| 2 | 1 | Suburban | Community care | 45 community care teams |
| 3 | 1 | Suburban | Community care | 45 community care teams |
| 4 | 2 | Urban | Nursing home | 17 locations for residential care |
| 5 | 3 | Suburban | Nursing home | 1 small-scale care location for private care |
| 6 | 4 | Rural | Community care | 34 community care teams |
| 7 | 1 | Suburban | Community care | 45 community care teams |
| 8 | 4 | Rural | Nursing home | 31 locations for residential care |
| 9 | 4 | Rural | Community care | 34 community care teams |
| 10 | 5 | Suburban | Nursing home | 2 locations for residential care |
| 11 | 4 | Rural | Community care | 34 community care teams |
| 12 | 1 | Suburban | Community care | 45 community care teams |
| 13 | 6 | Suburban | Community care | 35 community care teams |
| 14 | 6 | Suburban | Nursing home | 12 locations for residential care |
| 15 | 7 | Urban | Nursing home | 29 locations for residential care |
