# Supplementary material for: Certified nursing assistants’ perspectives on their role in advance care planning for older persons: A qualitative study
Source: Palliat Med. 2026 Jul 31;40(8):1158–67. doi: 10.1177/02692163261455222 (PMC13478406; doi:10.1177/02692163261455222)
Supplement: Supplementary file 3 [file sj-docx-3-pmj-10.1177_02692163261455222.docx]

### **Supplementary file 3. Interview guide**

##### Introduction

Thank you for participating in this study and taking the time for this interview. This study examines the role that nursing assistants have and/or wish to have in advance care planning for frail older people (brief explanation; more background will follow). This interview covers various themes, and we would like to know what your experiences are.

You may ask questions at any time. If you do not feel comfortable answering a question, you are not obliged to do so, please let us know. Your data will only be accessed by researchers within our research team. Additionally, your data will be encrypted, making it untraceable to you. This interview will last approximately 45 to 60 minutes.

##### Advance Care Planning (Current Role)

1. How would you define advance care planning?

- If you are unsure, what do you think it involves?

Explanation of the definition so that the interviewer and interviewee have the same understanding about advance care planning: *Advance care planning is internationally defined as “the ability of individuals to determine personal goals and preferences for future medical treatment and care, discuss them with loved ones and nursing assistants, and record and revise them as necessary.”^1^*

2. Do you often work with patients with palliative care needs?

- In practice, do you see a need for patients to discuss their wishes and goals?

3. How do you notice or hear this from patients?

- After observing these cues, how do you address them?
- What do you do with this information?
- Do you share this information with other caregivers? If yes, with whom?

In addition to responding to signals from clients, a conversation about advance care planning can be scheduled with the client, loved ones, and possibly other caregivers.

4. Do you have experience conducting an advance care planning conversation?

- Can you share how this is done?

5. How do you feel about having a conversation about advance care planning?

- To what extent do you feel comfortable doing this?
- What helps you to have such a conversation?
- What barriers do you experience in having such a conversation?

##### Capability

Engaging in a conversation about advance care planning requires certain knowledge and skills (examples: empathy, trust, being realistic regarding wishes).

6. What do you think is important to know or be able to do in order to play a role in this process?
7. Do you feel you have the right skills (knowledge) to play a (greater) role in advance care planning?

- Why or why not? (How did you acquire this knowledge? Through training or years of experience?)
- What barriers do you experience?
- What facilitators?
- How do you think this could be done differently?

### Opportunity

To play a role in advance care planning for vulnerable older people, it is important that you are involved in the process (receiving information, observing, ACP conversation).

8. As a nursing assistant, how would you ideally like to be involved in the initiation, implementation, and evaluation of advance care planning?
9. Do you feel you are currently given enough opportunities to play a (greater) role in advance care planning by other caregivers, for example?

- Why or why not?
- Do you find barriers or facilitators to this?
- How do you think this could be done differently?

##### Motivation

10. To play a role in advance care planning for vulnerable older people, it is important that you are motivated and willing to do so.
11. From your role as a nursing assistant, would you like to be more involved in advance care planning?

- Why or why not?
- Do you find barriers or facilitators to this?
- In your opinion, how could this be done differently?

12. What tasks would you personally like to have in the advance care planning process?

##### Summary

You have mentioned many barriers and supporting factors.

13. Could you briefly name the most important barriers for you? And the most important facilitators?

##### Closing

14. Are there any other things you would like to share that have not yet been addressed?
15. Do you have any questions?

Then we are at the end of the interview. Thank you again for participating in this interview and taking the time to do so. I have learned a lot from you, and I hope it was an enjoyable experience for you as well.

**References**

1.	Rietjens JAC, Sudore RL, Connolly M, et al. Definition and recommendations for advance care planning: an international consensus supported by the European Association for Palliative Care. *The Lancet Oncology* 2017; 18: e543–e551. DOI: 10.1016/S1470-2045(17)30582-X.
